# An Account of the North Kensington Friendly Workers among the Poor

**Published:** 1892-08-27

**Authors:** 


### THE CURE OF POVERTY,

### AN ACCOUNT OF THE NORTH KENSINGTON FRIENDLY WORKERS AMONG THE POOR.

The air is so full of schemes for helping the poor and eliminating poverty from the world; most of these schemes are so far-reaching, and their originators are so fully convinced that in them will be found the cure for the most persistent of modern maladies; that one feels diffident in laying before a world already surfeited with philanthropic fancies the details of an organization so modest as that of the North Kensington Friendly Workers among the poor. But this organisation has one claim on public attention which many of more ostensible importance had not. It succeeded in what it set out to do.

I hope to show how this was done, and how to do it in any district. But before going into details of working, I would like to draw attention to the name of the association—the North Kensington Friendly Workers among the Poor. The name is important; it contained a pledge on the part of all who aided in the work to maintain a certain attitude in their labours, a certain relation to those they laboured for, on which all hope of success depended.

First, then, the place where the work was done—North Kensington. When May Kendall makes her maid-of-all-work sing of "happy, happy Kensington" as a contrast to Drury Lane, I think she means South Kensington. There is poverty there, no doubt, but not to anything like the same extent as in the northern division. In North Kensington there are, besides the ordinary allowance of courts and mews hiding between the rows of large houses, long streets of small houses, suitable enough for a family in modest circumstances if only one such occupied them. But for the most part they are let out in flats or in single rooms to lodgers. Where the lodger is a single man or woman, a clerk or a shop-girl, or even a young married couple, one need not assume dire poverty; but when this room or two forms the home of a large and growing family, you may safely assume that money is too scarce to permit of removal to more comfortable quarters. In London, more than anywhere else, may you be sure of this. The Londoner likes comfort; he has even a turn for luxury. A Glasgow artisan will live contently in a "room and kitchen" till he has saved and invested enough to blossom forth in a suburban villa and a servant. You cannot judge there of a man's income by the manner in which he lives. He may be far richer than you guess. In London it is not so; rather, if you are deceived, will it be in an opposite direction, by the presence of little ornaments, evidences of money spent otherwise than on necessaries, which to the casual eye imply a certain superfluity of gold. This appearance is misleading, as misleading as the long feathers in the hats of East-end factory girls, or as the piano in the room of a seamstress whom I knew. She kept that, though she had been forced to sell her bed, and did not fare sumptuously every day. I sometimes think there are a good many such feathers and pianos in the imposing North Kensington streets and squares. Take it all round, villas and terraces, courts and lanes, and little streets with wonderful long names, North Kensington is not a rich locality. The district looked after by the association contains 1,934 houses, over 1,000 of which contain poor people; that means that, speaking roughly, one half of the population of North Kensington may any day require help from the other half.

What was needed from the other half was then—to go on with our title—that they should be Friendly Workers. The other half has not, unfortunately, seen so clearly as might be desired its duty in this matter. The other half never does; only one-tenth of it at the most can ever be persuaded to be a real friend to the poor, a real worker among the poor; the other nine-tenths talk languidly about referring such matters to the proper agencies, without troubling to inquire by whom these agencies are established, maintained, and worked. The friendly worker among the poor was the man or woman who would find out these agencies, see what they were suitable for, apply them where possible to the case in hand, and failing that, make use of such personal means or interest as might raise the case from the category of mere "cases" into that of independent self-supporting human beings. The worker must be a friend, to whom the poor man and woman can unaffectedly confide every anxiety and trouble that weighs them down, a friend who will be interested in things which do not call for what poor-law authorities term in their limited phrase "relief"; a friend whose advice will be as carefully given, as reliable, and as valuable as his money. To attain this the friend must be a worker; not a mere giver of doles. The friend must give time and energy to the investigation of every case; to finding out what work his poor friend is fit for, and to obtaining that work for him. The friend must look to the well-being of children, when the demands of labour compel them to be separated from their parents. He must try to arrange that the care of aged parents shall not too much interfere with the work of the children who support them. He must find means to make the poor help themselves; he may make things easier for a large circle if he puts them in the way of helping each other.

To exemplify the sort of thing which a Friendly Worker must undertake, let me take one of the many cases given in the association's last report:—

Case of a man whose wife died from negligence after child birth.—The man was left with three children (girls), aged respectively eight years, seventeen months, and eighteen days. We found him in a most hopeless condition, not knowing how to have the children looked after, so as to allow him to seek for work, which he was out of at the time. We made arrangements at once for a woman living in the same house to take charge of the children during the day time, until we found a more permanent foster mother. In a few days we arranged with another woman to act as foster mother to the children, but she found that the second child was suffering from consumption of the bowels and she then refused to take them, so that our arrangements with the first woman had to be extended for a time. We had the child examined by a doctor, who ordered it to a hospital, where it was then taken, but refused admittance on the grounds that it only required proper care at home. As under the circumstances this was a difficulty we told the father to take the child down at once to the relieving officer, in order to get an admission order to the workhouse infirmary. The result of this was that the child was admitted within sixteen days of the mother's death. Whilst the father was out of work we helped the case at first in conjunction with St. Columb's Church by paying for the keep of the children and afterwards procured from the Campden Charities, after making full statement of the case to them, the sum of £5 to be expended in making an allowance of 10s. a week, towards the support and proper nourishment of the children. This help was most useful, and £2 was used in this way, when the man got permanent work and was then able to support the family himself. We had by this time got the children comfortably settled in a respectable family, the father living in the same house. We were able in this case to return £3 to the Campden Charities and to assure them of the great good done by their grant. We were afterwards instrumental in getting the man £6 from the War Office, being money which had belonged to a deceased soldier brother, but which he had been unable to get owing to a difficulty in proving next of kinship, the brother having enlisted under a false name. This money was divided between the man and his two sisters.

This case shows a variety of incidents in which ordinary modes of help—worked by "machinery" with little help from human heart or brain—would have been useless. The man was in such a plight as befalls most men in such circumstances, whether they be rich or poor. One may have the most remunerative work to do, but money would not settle the question of how the children are to be looked after. And when there is no money to be had if the children are looked after, and can only be had if those on whom one would most willingly spend it are left neglected, what then? Then the friend came in, took all manner of pains to see that the children were properly cared for, bringing up another plan, and yet one more, when the first had failed; freeing the widowed father to seek for work and to do it, obtaining money to support all till he should begin to earn wages, securing his share of money due to him which he knew not how to claim, and then—note this, please—returning what was left of the money advanced, as soon as the help it was meant to give had succeeded in its object, to the charities which had given it. I should like to know what was the feeling of the managers of the Campden Charities when that £3 was returned to them. It was probably an unique experience. I should fancy they said something like this: "These Friendly Workers evidently know what they are doing. They don't throw away money recklessly. We don't mind entrusting them with some again."

What would have happened if this case had been treated by machinery? The £5 given by the Campden charities would have been spent, then the children would have been taken to the workhouse, and the family, with all its pure affections, its elevating sense of duty, would have been destroyed. We should have had instead only three paupers and an irresponsible man—but for the intervention of the North Kensington Friendly Worker.

I have taken this case out of its due order because it shows so clearly the circumstances in which something beyond the ordinary methods of charity is required, something that can combine various kinds of help, something that can discriminate as well as bestow, something that demands a friend as well as a donor. With such elucidation as a specimen case can give, I proceed to tell the history and methods of the North Kensington Friendly Workers.

At a conference of charitable workers, held at All Saints' Schools, Kensington, on the afternoon of Saturday, July 27th, 1889, Mr. Henry C. Burdett proposed a scheme for the better employment of the charitable funds and societies existing in North Kensington. These were to be extended where needed, but the chief aim was to develop the utmost usefulness of existing organisations. Mr. Burdett had studied the Associated Charities of the city of Boston, U.S.A., and found in the Boston system the idea which, with the modifications required by English life, he wished to introduce into North Kensington. Mr. Burdett laid down as a first principle that alms, though a necessary part, are not the whole of charity. If you replace throughout the whole of the xiii. chapter of the First Epistle to the Corinthians, the word "charity" by the word "alms," you will see that in a moment. Charity, said Mr. Burdett, must do four things:—

I. Relieve worthy need promptly, fittingly, and tenderly.
II. Prevent unwise alms to the unworthy.
III. Raise into independence every needy person, where this is possible.
IV. Make sure that no children grow up to be paupers.

Relief, detection, elevation, and prevention are all essential parts of a complete system.

Of the first of these objects nothing need be said; all Christian men and women, all who have a human soul, are agreed in this. It is necessary, and it is comparatively easy. To avoid the giving of unwise alms to the unworthy is infinitely more difficult. In the first place, those who least deserve alms are the most ready to seek them, the most fluent in their stories of distress, the most effusively grateful for help bestowed. Private individuals giving help independently of agencies are the most likely to be so misled. There is an appeal to their personal sympathy, their capacity for perceiving real distress, above all to the pain which they themselves would feel if compelled to confess all their troubles to the unsympathetic officer of a society—pleas adapted to the vanity of the philanthropist and the refinement of his nature, which rarely fail of their effect. One hesitates to calculate how much money is thus mis-spent in London every year: still more unwilling would one be to reckon up the mischief that it does. Money that does not do good does harm. When it does not help people to climb out of the mire, it plunges them deeper into it; when it does not set them on the high road to independence, it makes them confirmed paupers.

Now, this can only be prevented by intercourse between charitable workers. It is well known that some of these clever schemers professedly belong to two or three churches, often enough of different denominations, and get aid from all. If these workers could only meet and compare notes much might be saved from these professional "sorners" (a Scotch word, signifying those who habitually live at the expense of another, for which I know no English equivalent), and would be available for more worthy objects. The first thing necessary, then, for the successful administration of the charities of any district is complete co-operation between all the donors and philanthropic workers in that district. In cases which they had proved to be worthy of help they could supplement each other's efforts; in unworthy cases each could prevent the other being imposed upon. Besides, although most of us have the power to help some of our fellow creatures, we may not be so situated as to help all those who apply to us for aid. Suppose that a bricklayer out of work asks help from a private lady of means, what can she do for him? She can give food which, when eaten, and clothes which, when worn, leave the man and his family exactly where they were before. She does not permanently improve their condition by her gifts; rather, if she keeps up her alms for long, will she tend to pauperise them. Human nature is not ideal, and if a man finds out that he can be kept without working, it is ten to one that he will remain idle. Would it not be infinitely better if the well-meaning, but practically impotent, lady was acquainted with the builder in the next street who could give the bricklayer work? It would cost the lady less, except in time and trouble; but it would be an infinitely more valuable form of help. Cost is not inevitably equivalent to value. Carlyle declares that the importation and free distribution of thousands of horses into any country would do less for its inhabitants than the invention of the modest wheelbarrow. In all our schemes let us seek for the wheelbarrow.

(*To be continued.*)

